# A Null Mutation of *TNFRSF11A* Causes Dysosteosclerosis, Not Osteopetrosis

**DOI:** 10.3389/fgene.2022.938814

**Published:** 2022-06-24

**Authors:** Tarık Kırkgöz, Behzat Özkan, Filiz Hazan, Sezer Acar, Özlem Nalbantoğlu, Beyhan Özkaya, Melike Ataseven Kulalı, Semra Gürsoy, Shiro Ikegawa, Long Guo

**Affiliations:** ^1^ Division of Pediatric Endocrinology, Dr. Behçet Uz Children’s Education and Research Hospital, Izmir, Turkey; ^2^ Department of Medical Genetics, Dr. Behçet Uz Children’s Education and Research Hospital, Izmir, Turkey; ^3^ Division of Pediatric Genetics, School of Medicine, Afyon Kocatepe University, Afyonkarahisar, Turkey; ^4^ Division of Pediatric Genetics, Dr. Behçet Uz Children’s Education and Research Hospital, Izmir, Turkey; ^5^ Laboratory for Bone and Joint Diseases, RIKEN Center for Integrative Medical Sciences, Tokyo, Japan

**Keywords:** TNFRSF11A/TNR11/RANK, dysosteosclerosis, sclerosing bone dysplasia, osteopetrosis, mutation

## Abstract

Dysosteosclerosis (DOS) is a rare sclerosing bone dysplasia characterized by unique osteosclerosis of the long tubular bones and platyspondyly. DOS is inherited in an autosomal recessive manner and is genetically and clinically heterogeneous. To date, four individuals with DOS who have five different *TNFRSF11A* mutations have been reported. Based on their data, it is hypothesized that mutations producing aberrant mutant RANK proteins (missense or truncated or elongated) cause DOS, while null mutations lead to osteopetrosis, autosomal recessive 7 (OPTB7). Herein, we present the fifth case of *TNFRSF11A*-associated DOS with a novel homozygous frame-shift mutation (c.19_31del; p.[Arg7CysfsTer172]). The mutation is predicted to cause nonsense mutation-mediated mRNA decay (NMD) in all RANK isoform transcripts, resulting in totally null allele. Our findings suggest genotype-phenotype relationship in *TNFRSF11A*-associated OPTB7 and DOS remains unclear, and that the deficiency of *TNFRSF11A* functions might cause DOS, rather than osteopetrosis. More data are necessary to understand the phenotypic spectrum caused by *TNFRSF11A* mutations.

## Introduction

Dysosteosclerosis (DOS) is a rare form of dense bone disease characterized by osteosclerosis and platyspondyly (Spranger et al., 1968). Its features are short stature, recurrent fractures, optic atrophy, cranial nerve palsy, developmental delay, flattened fingernails, skin related complications, and failure of tooth eruption (MIM %224300). Irregular osteosclerosis, flattened diffusely dense vertebral bodies, sclerotic skull, radiolucent sub-metaphyseal portions of the long tubular bones with sclerotic diaphysis are radiological features of DOS (Houston et al., 1978; Elçioglu et al., 2002; Whyte et al., 2010; Guo et al., 2018). Moreover, metaphyseal osteosclerosis and platyspondyly are the characteristic and important guiding findings in differentiating DOS from other types of sclerosing bone dysplasia (Howaldt et al., 2019).

DOS is inherited in an autosomal recessive manner and is genetically and clinically heterogeneous. Four disease genes for DOS have been described to date (Campeau et al., 2012; Guo et al., 2018; Guo et al., 2019; Howaldt et al., 2019; Uludağ Alkaya et al., 2021). *SLC29A3* (solute carrier family 29 member 3) was the first identified gene, followed by *TNSFR11A* (tumor necrosis factor receptor superfamily member 11a; also known as RANK (receptor activator of nuclear factor kappa B)), *TCIRG1* (T cell immune regulator 1) and *CSF1R* (colony stimulating factor 1 receptor) (Campeau et al., 2012; Guo et al., 2018; Guo et al., 2019; Howaldt et al., 2019; Uludağ Alkaya et al., 2021). Notably, *TNSFR11A* and *TCIRG1* mutations are also reported in osteopetrosis, autosomal recessive 7 (OPTB7) and osteopetrosis, autosomal recessive 1 (OPTB1), respectively (Frattini et al., 2000; Guerrini et al., 2008; Pangrazio et al., 2012).

To date, four DOS individuals with five different *TNFRSF11A* mutations (2 missense/nonsense, 2 splice-site, 1 deletion) have been reported (Guo et al., 2018; Xue et al., 2019; Xue et al., 2020; Xue et al., 2021a; Xue et al., 2021b). According to the previous hypothesis for genotype-phenotype relationship, aberrant mutant RANK proteins (missense or truncated or elongated) cause DOS, while null mutations lead to OPTB7 (Guo et al., 2018; Xue et al., 2019; Xue et al., 2020; Xue et al., 2021a). Herein, we present the fifth case of *TNFRSF11A*-associated DOS in a 19-month-old boy, in whom we identified a novel homozygous mutation in *TNFRSF11A* (c.19_31del; p.[Arg7CysfsTer172]). Unlike the previously reported DOS mutations, the mutation is predicted to cause nonsense mutation-mediated mRNA decay (NMD) in all RANK isoform transcripts, which leads us to re-consider the phenotype and genotype relationship of the *TNFRSF11A* mutation.

## Materials and Methods

### Case Report

A 19-month-old boy was consulted to pediatric endocrinology unit for hypocalcemia and short stature. He was the third baby to healthy consanguineous Turkish parents who had healthy boys and two abortions. Family history was unremarkable with no affected family members. The prenatal course was uneventful. The proband was born at term by normal Cesarean section with a birth weight of 3,200 g (+0.02 standard deviation score (SDS)). He had a history of hypocalcemic convulsion at day 4, and was treated with intravenous calcium and vitamin D supplementation. The patient was discharged to home with phenobarbital therapy at day 14. From 4^th^ month, he was hospitalized three times due to lower respiratory tract infection.

On the physical examination at age 19 months, his height, weight and head circumference were at −3.1, −2.7, and +0.2 SDSs, respectively. Midface hypoplasia, edematous eyelids, down slanting palpebral fissures, long eyelashes, long philtrum, high arched palate, low set ears, and micrognathia were detected. A prominent forehead, open anterior fontanelle (4 × 4 cm), pectus carinatum, café-au-lait spot (4 × 3 cm) on anterior thorax, and bowing of femora and ulnae were noticed. Ophthalmological examination revealed optic atrophy and horizontal nystagmus. Moderate delay in developmental milestones was observed.

**FIGURE 1:**
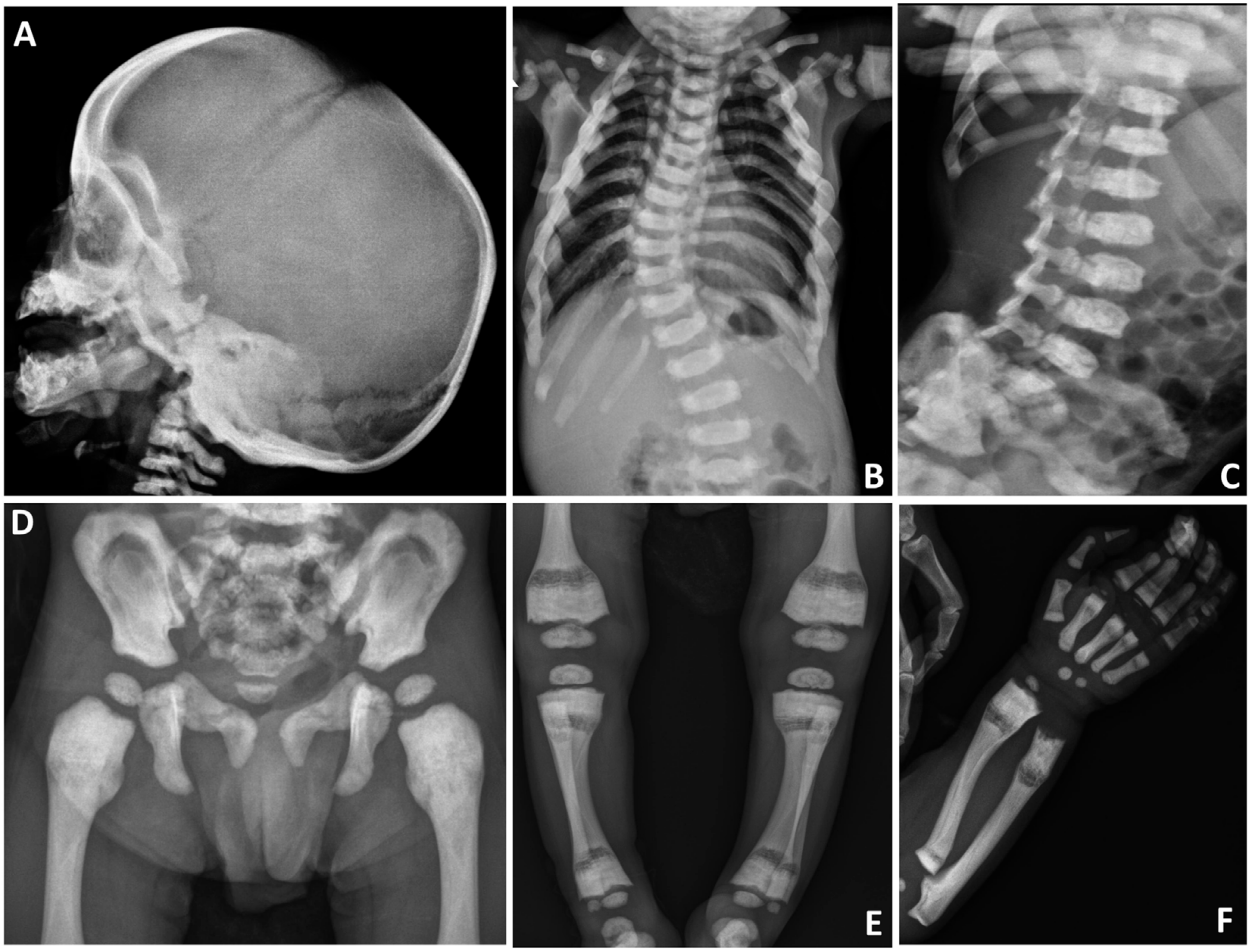
Skeletal survey of the patient. **(A)** Sclerosis of the craniofacial bones, especially at the skull base. **(B)** Scoliosis at the thoracic level. **(C)** Plate irregularities and mildly reduced height of the vertebral corpus (platyspondyly). **(D)** Sclerosis at the pelvic bones, especially in the iliac bodies. **(E)** Radiolucency at the metadiaphyseal junction and sclerosis at the end of long bones. **(F)** Sclerosis of the radius, ulna, metacarpal bones, and proximal phalanges. Radiolucency at the metadiaphyseal junction of radius and ulna is also noted.

On biochemical evaluation, serum calcium was 7.4 mg/dl (Normal (N): 9.0–11), phosphorus was 4.4 mg/dl (N: 4–6.5), ALP was 84 U/L (N: 116–450), albumin was 4.1 g/L (N: 3.5-5.5), PTH was 102.3 ng/L (N: 15–88), and 25-OH vitamin D was 27 μg/L (N: 30–100). Liver-kidney function tests, thyroid function tests and ions were normal. Skeletal survey showed diffuse osteosclerosis of the craniofacial, axial and appendicular skeletons, especially in diaphyseal areas of the long tubular bones (Figures 1A,D–F). In the evaluation of the vertebral structures, end plate irregularities, mildly reduced height (platyspondyly) and thoracic and lumbar scoliosis were observed (Figures 1B,C). Pelvic bones showed sclerosis, especially in the iliac bodies (Figure 1D). Calcium replacement (50 mg/kg/day) and maintenance dose vitamin D (400 U/day) treatments were started, and the calcium value was normalized within a week. After normalization of serum calcium level, the treatment was discontinued and biochemical parameters remained normal in the follow-up. After his clinical improvement at age 21 months, he was discharged to follow up in the outpatient clinic.

One month after the discharge, he was hospitalized to the pediatric intensive care unit of another hospital with complaints of fever, cough, vomiting, and weight loss. In the initial evaluation, lower respiratory tract infection and sepsis were considered and appropriate fluid and antibiotic therapy was initiated. When respiratory findings worsened and respiratory acidosis became evident, he was intubated and connected to a mechanical ventilator. Cranial magnetic resonance imaging revealed severe hydrocephalus and therefore a ventro-peritoneal shunt was inserted. Despite the treatments, his clinical condition deteriorated and he died after 4 months of follow-up in the pediatric intensive care unit at age 26 months.

### Targeted Sequencing

After written informed consents, blood samples were obtained from the patient, parents, and brothers. Genomic DNA was extracted from leukocytes using the MagPurix kit (Zinexts Life Science Corp., New Taipei City 235, Taiwan), according to manufacturer’s instructions. For the molecular genetic evaluation, a Custom Target Capture-based Osteopetrosis gene panel (Celemix Inc., Seoul, Korea), which was designed according to the before-2018 ENMC classification was used.

### PCR and Sanger Sequencing

The variant identified by the targeted sequencing was confirmed by Sanger sequencing. The region of genome including the mutations was amplified by PCR and sequenced both strands. Exon-specific Sanger sequencing was performed using 5′-GAG​CTT​GGG​CAC​CAC​CTG-3′ and 5′-TCC​GCT​CCC​CAA​AAC​TCC-3′ primers on Applied Biosystems 3500 Genetic Analyzer. The sequences were evaluated by two different sequencing programs; i.e., CLC Genomics Workbench 3 sequencing program (Qiagen) and Chromas lite Software (Technelysium, South Brisbane QLD, Australia).

### Variant Evaluation

The variant was evaluated by dbSNP (http://www.ncbi.nlm.nih.gov/projects/SNP/), 1000 genomes (http://www.1000genomes.org/), ExAC (http://exac.broadinstitute.org/), gnomAD (http://gnomad.broadinstitute.org/), ESP6500 (http://evs.gs.washington.edu/EVS/), Human Gene Mutation Database (HGMD; http://portal.biobase‐international.com/hgmd/pro/start.php), and Mutation Taster (http://mutationtaster.org).

## Results and Discussion

**FIGURE 2:**
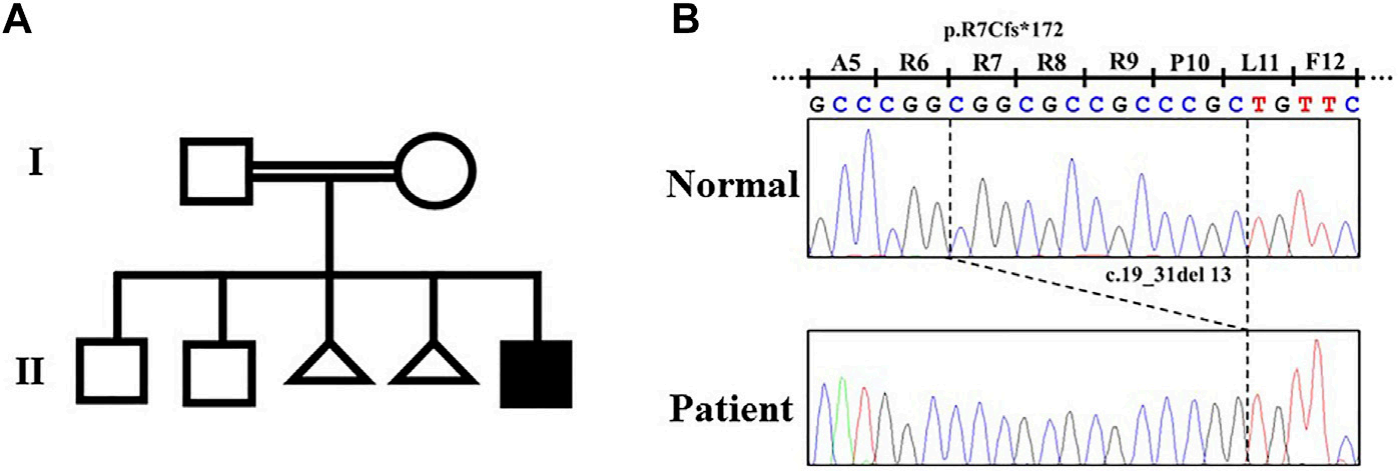
Pedigree of the family with dysosteosclerosis and the *TNFRSF11A* variant. **(A)** Pedigree. **(B)** Electropherograms of the Sanger sequence analysis.

By the target sequence, a novel homozygous frameshift variant (NM_003839.3: c.19_31del; p.Arg7CysfsTer172) in *TNFRSF11A* was detected. The variant was confirmed by Sanger sequencing (Figure 2). The parents were both heterozygous for the variant (Figure 2). This variant was interpreted as “Likely Pathogenic” according to American College of Medical Genetics criteria (ACGM) (Richards et al., 2015). This variant did not exist in any available databases including dbSNP, 1000 genomes, ExAC, gnomAD, ESP6500, and HGMD. According to an *in silico* analysis with Mutation Taster, this variant was predicted to affect signal peptide and protein structure, and causes NMD.

**FIGURE 3:**
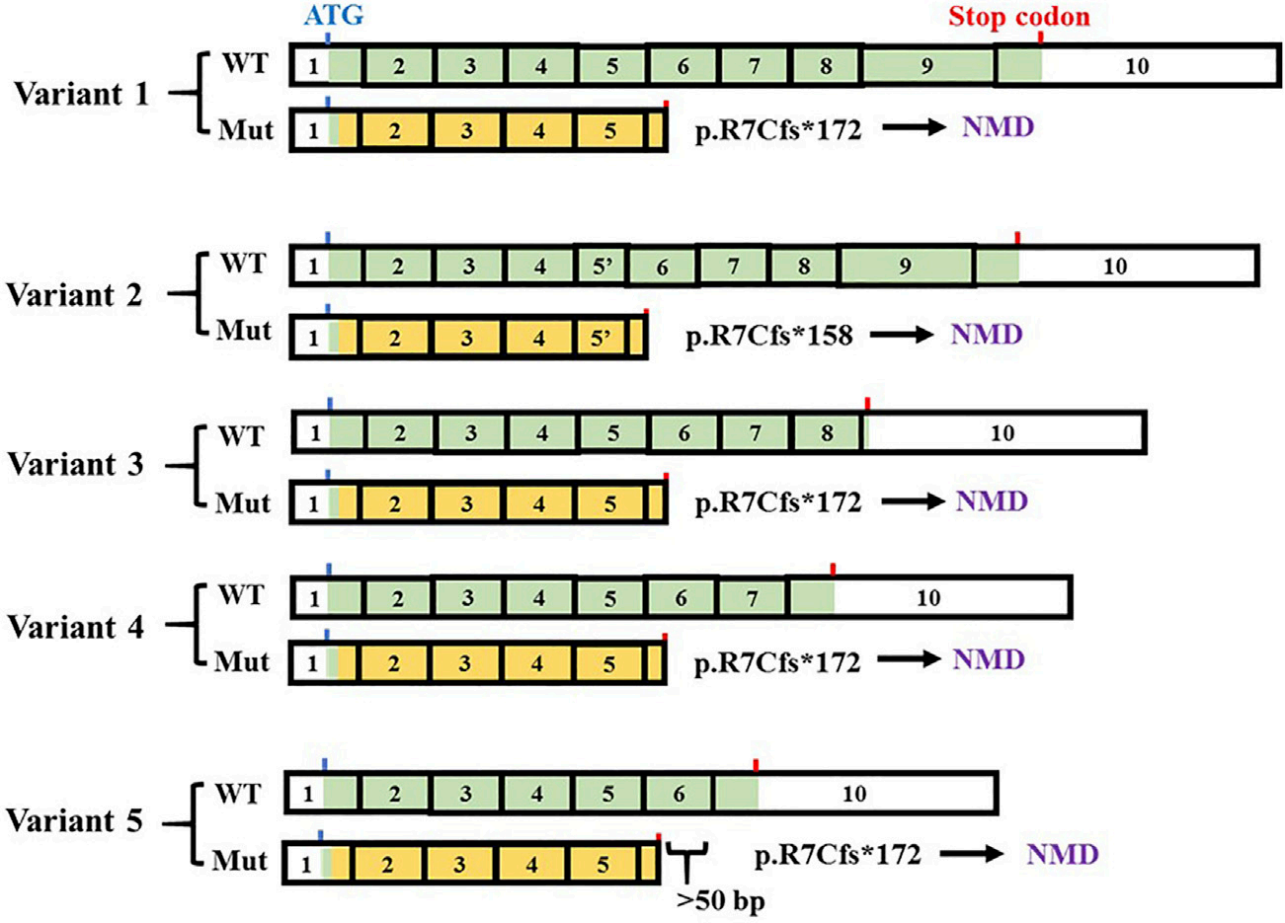
TNFRSF11A isoforms and the effect of the variant (c.19_31del). Five variant transcripts are in NCBI database (variant 1: NM_003839.3; variant 2: NM_001278268.1, variant 3: NM_001270949.1, variant 4: NM_001270950.1, variant 5: NM_001270951.1). The exons are numbered based on the longest variant (variant 1). The changed parts of coding sequence are labeled in orange. The positions of the first ATG and the stop codons were indicated by blue and red bars, respectively. c.19_31del produces no transcripts due to nonsense mutation-mediated mRNA decay (NMD).

DOS and OPTB7 are inherited as autosomal recessive traits and are clinically very similar. However, they can be differentiated by the remarkable radiological features of DOS, including platyspondyly and enlarged lower metaphyseal parts of tubular bones with punctate densities and radiolucency (Spranger et al., 1968; Houston et al., 1978; Elçioglu et al., 2002; Whyte et al., 2010; Guo et al., 2018). OPTB7 is caused by loss-of-function mutations of *TNFRSF11A*, which are missense mutations or deleterious mutations leading to NMD (Guerrini et al., 2008; Pangrazio et al., 2012; Xue et al., 2021b). *TNFRSF11A*-associated DOS is caused by splice-site or frameshift mutations which are capable of producing concurrent truncated or long RANK proteins (Table 1). Five *TNFRSF11A* isoforms encoding five different proteins have been identified (Figure 3). It has been speculated that the clinical difference of DOS and OPTB7 may relate to the different effects of *TNFRSF11A* mutations in different *TNFRSF11A* isoform transcripts (Guo et al., 2018). The variants identified in the first three reported cases of *TNFRSF11A*-associated DOS cause NMD in some transcript isoforms, while simultaneously producing truncated or elongated RANK proteins in the remaining transcript isoforms according to the results of RT-PCR for the patient-derived cells and the exon trapping assay for cell lines (Guo et al., 2018; Xue et al., 2019; Xue et al., 2020). The functions of these truncated or elongated RANK proteins remain unclear. In a mutant mouse model with a nine-amino-acid insertion in *Tnfrsf11a*, the homozygotes develop osteopetrosis while the heterozygotes show osteolytic lesions. The abnormal RANK proteins in the mutant mice accumulated in Golgi apparatus and increased osteoclastogenesis by activating the unfolded protein response (Alonso et al., 2021). The findings suggest that the truncated or elongated RANK proteins generated in the first three cases of *TNFRSF11A*-associated DOS probably results in gain-of-function, which would be associated with the DOS phenotype. However, in the fourth case, a single amino acid substitution in *TNFRSF11A* was identified, suggesting that phenotype-genotype association is not easily predictable in *TNFRSF11A*-related bone diseases (Table 1) (Xue et al., 2021a).

**TABLE 1:** Clinical and radiographic findings of *TNFRSF11A*-associated dysosteosclerosis.

	Case				
	1^st^ ^a^	2^nd^ ^b^	3^rd^ ^c^	4^th^ ^d^	5^th^ ^e^
Mutation^f^					
Allele 1 (mutant protein)	c.616+3A > G (p.N174Kfs*31)	c.784G > T (p.E262_Q279del)	c.1664del (p.S555Cfs*12)	c.385C > T (p.R129C)	c.19_31del (none)
Allele 2 (mutant protein)	c.616+3A > G (p.N174Kfs*31)	c.784G > T (p.E262_Q279del)	c.414_427 + 7del (none)	c.385C > T (p.R129C)	c.19_31del (none)
Clinical data					
Age at onset	3 years	17 years	8 months	13 months	19 months
Height [cm]	150 (−1.98 SD)	159.8 (−1.90 SD)	81 (−3.7 SD)	115 (−7.35 SD)	70.5 (−3.1 SD)
Cranial nerve palsy	−	−	+	+	+
Hydrocephaly	−	−	−	+	+
Hepatosplenomegaly	−	−	−	+	−
Hypogammaglobinemia	−	−	−	−	−
Mandibular or maxillary osteomyelitis	−	+	−	+	−
Anemia	−	+	NA	+	−
Thrombocytopenia	−	NA	NA	+	−
Extramedullary hematopoiesis	−	−	−	+	−
Indication for HSCT	−	−	+	−	−
Radiographic data					
Platyspondyly	+	+ mild	+ mild	+	+ mild
Concaved vertebrae at posterior thirds	+	+	-	-	-
Radiolucency of widened submetaphyseal portions of the tubular bones	+	+	+	+	+

HSCT, hematopoietic stem cell transplantation; NA, not available.

a Guo et al. J Hum Genet 2018.

b Xue et al. J Bone Miner Res 2019.

c Xue et al. J Hum Genet 2020.

d Xue et al. J Hum Genet 2021.

e This study.

f Mutations are named according to NM_003839.3; The longest mutant proteins among the isoforms are listed.

The variant we identified in this study leads to frameshift and premature termination in exon 6 (Figure 3), thus being predicted to be a null mutation due to NMD. Contrary to the previous hypothesis, the clinical findings of our patient were quite compatible with DOS, while the variant causes NMD in all transcript isoforms (Figure 3). These conflicting findings suggest that the relationship between genotype and phenotype in *TNFRSF11A*-associated DOS cases is complicated and further studies are needed. It would also be interesting to explore the similar issue further for *TCRIG1* (Howaldt et al., 2019).

Our study reports the first case of DOS caused by *TNFRSF11A* null mutations, which are previously considered to cause OPTB7. We re-evaluated the phenotypes of the OPTB7 patients with null mutations. Until now, six patients carrying seven mutations causing NMD in all or some of the transcripts have been reported (Table 2) (Guerrini et al., 2008; Pangrazio et al., 2012; Silveira et al., 2021; Xu et al., 2021). Their radiographic data critical to differentiate DOS are not publicly available, except for a case reported by (Xu et al., 2021). Its skeletal phenotype is more compatible with the diagnosis of DOS rather than OP, since the platyspondyly with concaved vertebrae at posterior thirds and the radiolucency of widened sub-metaphyseal portions of the tubular bones are evident (Xu et al., 2021). Based on the findings on the case and our case, it could be hypothesized that the deficient *TNFRSF11A* functions causes a broad phenotypic spectrum covering DOS and OP. The remaining RANK function of the *TNFRSF11A* mutations may be an important factor that decides the radiographic features of the patients.

**TABLE 2:** Patients with null mutations of *TNFRSF11A*.

Reference	Patient	Mutation		Position of New stop codon^b^	Isoform^c^					X-ray
		Nucleotide^a^	Type		1	2	3	4	5	
Guerrini et al.	P1	Allele 1 = 2: c.838G > T	Nonsense	exon 9	–	–	WT	WT	WT	NA
Am J Hum Genet 2008	P2	Allele 1 = 2: c.1301G > A	Nonsense	exon 9	–	–	WT	WT	WT	NA
Pangrazio et al. J Bone Miner Res 2012	P3	Allele 1: c.247G > T	Nonsense	exon 3	–	–	–	–	–	NA
		Allele 2: c.372C > A	Nonsense	exon 4	–	–	–	–	–	
	P4	Allele 1 = 2: c.328dupC	Frame-shift	exon 4	–	–	–	–	–	NA
Xu et al. BMC Surg 2021	P5	Allele 1 = 2: c.1196C > G	Nonsense	exon 9	–	–	WT	WT	WT	A
Silveira et al. Am J Med Genet C 2021	P6	Allele 1 = 2: c.1371_1372delTG	Nonsense	exon 9	–	–	WT	WT	WT	NA
This study	P7	Allele 1 = 2: c.19_31del	Frame-shift	exon 6	–	–	–	–	–	A

a Mutations are named according to NM_003839.3; the longest transcript corresponding to isoform 1.

b Exon 10 is the last exon (NM_003839.3).

c indicates that no isoform is expected to be produced due to the nonsense mutation mediated RNA decay.

WT, wild type; NA, not publicly available; A, publicly available.

The previous four cases of TNFRSF11A-associated DOS show that the skeletal phenotypes are heterogeneous even if all cases meet the criteria of DOS. Generally, the young cases (Case 3 and 4) had a clear radiolucent band in the sub-metaphyseal region of tubular bones, which was widely splayed and sclerotic (Xue et al., 2020; Xue et al., 2021a). In contrast, the radiolucency in the enlarged lower metaphyseal parts was diffuse in the older cases (Case 1 and 2) (Guo et al., 2018; Xue et al., 2019). Moreover, although platyspondyly were found in all cases, concaved vertebrae at posterior thirds were present only in the older cases (Table 1) (Guo et al., 2018; Xue et al., 2019). In this study, our patient at age 19 months showed similarities to the previous young cases in both spinal and sub-metaphyseal changes (Figures 1C,E; Table 1). These results suggest the phenotypes of *TNFRSF11A*-associated DOS considerably evolve with age and form a continuous phenotypic spectrum. Long-term observation would contribute to the understanding of the evolving phenotypes.

In conclusion, our findings indicate that we are still far from establishing a genotype-phenotype relationship in *TNFRSF11A*-associated OPTB7 and DOS. Detailed and continuous evaluation on the radiographic data remains necessary to elucidate the phenotypic spectrum caused by *TNFRSF11A* mutations.

## Data Availability

The datasets presented in this article are not readily available because study participants did not give full consent for releasing individual genomic data publicly. Requests to access the datasets should be directed to the corresponding authors.
